# The Effects of Syntactic Awareness to L2 Chinese Passage-Level Reading Comprehension

**DOI:** 10.3389/fpsyg.2021.783827

**Published:** 2022-02-01

**Authors:** Jing Zhou

**Affiliations:** Pomona College, Claremont, CA, United States

**Keywords:** syntactic awareness, L2 Chinese reading comprehension, grammatical judgment and correction test, word order test, word order, CSL (Chinese as a second language)

## Abstract

This study investigated the association between syntactic awareness and L2 Chinese passage-level reading comprehension among 209 Chinese as a second language adult-learners. The participants were administered a character knowledge test, a vocabulary knowledge test, a morphological awareness test, a grammatical judgment and correction test, a word order test, and two reading comprehension tests (multiple-choice questions and cloze test). Partial correlation analyses showed that the participants’ performance in two syntactic awareness tasks were significantly positively correlated with their passage-level reading comprehension. Multiple regression analyses revealed that syntactic awareness made a unique contribution to L2 Chinese reading even when the effects of age, major, gender, length of learning Chinese, character knowledge, vocabulary knowledge, and morphological awareness were controlled for. In addition, the word order knowledge had a stronger predicting power to L2 Chinese reading comprehension compared to the grammatical judgment/correction ability.

## Introduction

Syntactic awareness refers to an understanding and application of the grammatical structure of the language (Tunmer and Hoover, 1992). It has been found to contribute significantly to children and adults’ performance in word decoding, word reading, and text comprehension (e.g., Plaza and Cohen, 2003; Muter et al., 2004; Nation and Snowling, 2004). Recent research in both L2 English and L1 Chinese reading (Guo et al., 2011; Zhang, 2012; Yeung et al., 2013; Tong and McBride, 2017; Tong et al., 2021; Zhao et al., 2021a) has demonstrated the significant role syntactic awareness was playing in reading comprehension. However, few empirical studies have been carried out on the role of the syntactic skills in L2 Chinese reading comprehension among adult learners. The present study aimed to address this gap in the literature.

## Literature Review

There is growing evidence showing that readers’ performance in reading comprehension is significantly influenced by their degree of sensitivity to syntactic knowledge (e.g., Barnitz, 1980; Cain, 2007; Guo et al., 2011; Zhang, 2012; Tong et al., 2014; Hung, 2021; Sarbazi et al., 2021). This line of research usually investigated several predictors and examined the unique contribution of syntactic awareness to reading comprehension after other variables such as vocabulary knowledge and morphological awareness have been controlled for. In L1 English reading, Guo et al. (2011) examined the interrelations among vocabulary knowledge, morphological awareness, syntactic awareness, and reading comprehension among 151 undergraduate and graduate native English-speaking students enrolled at a public university. The study revealed that syntactic awareness predicted reading comprehension directly and indirectly through the mediation of vocabulary knowledge.

Similarly, recent evidence in reading in Chinese has also shown that syntactic awareness plays a role in reading in Chinese among native children (e.g., Yeung et al., 2013; Tong et al., 2014; Zhao et al., 2021b). Yeung et al. (2013) constructed a model of reading comprehension among 248 Chinese elementary school fourth grade children. Syntactic awareness was measured using two tests: a morphosyntactic knowledge test designed to measure the children’s ability to detect and correct morphosyntactic errors in sentences and a word order test where children were asked to arrange sentence segments in the order that they deemed correct. The study found that syntactic awareness, discourse skills, and verbal working memory had significant direct effects on reading comprehension. In Zhao et al. (2021a) study, syntactic knowledge was found to be both directly and indirectly related to reading comprehension *via* inference making, comprehension monitoring, and word reading among 164 Chinese third-grade students. Syntactic knowledge was measured using a conjunction cloze task. After controlling for word reading, verbal working memory, and discourse skills, syntactic awareness remained a unique predictor of L1 Chinese reading comprehension. In Chik et al.’s (2012) study, however, morphosyntactic knowledge, but not word order knowledge, was a unique contributor to reading comprehension. Some other studies measured syntactic awareness using a conjunction pair test (e.g., Tong et al., 2014). Tong et al. (2014) found that the conjunction cloze task was moderately correlated with previous and concurrent years’ discourse-level reading comprehension (*r* = 0.53 and 0.56, respectively). The grammatical judgment/correction task was also significantly correlated with previous and concurrent years’ reading comprehension (*r* = 0.19 and 0.18, respectively). The syntactic tasks together explained a small portion (2.3%) of the total variance of reading comprehension. The study concluded that children’s syntactic knowledge, especially in the use of conjunction words, appeared to be uniquely linked to discourse-level reading comprehension.

Most studies in reading in Chinese have been conducted among native Chinese children, and little is known about the role of syntactic awareness in reading in Chinese as a foreign or second language among adult learners (CFL/CSL). Moreover, previous studies on the effect of syntactic awareness have not been consistent. Some studies (e.g., Chik et al., 2012) suggested that morphosyntactic knowledge, but not word order knowledge, was a significant predictor of L1 Chinese reading, whereas other studies (e.g., Tong et al., 2014) found that conjunction words knowledge, not morphosyntactic knowledge, was a unique predictor. Thus, the question remains on how to measure L2 Chinese syntactic awareness to ensure content validity. Thirdly, no previous research has been carried out in L2 Chinese reading to examine how different tasks of syntactic awareness are associated with how L2 reading comprehension is measured. Therefore, this paper explores how word order knowledge and grammatical judgment/correction are associated with two types of reading comprehension tasks: the multiple-choice questions test, and the cloze test. It is hoped that this research will generate fresh insights into the role of syntactic awareness in L2 Chinese reading.

## Features of Chinese Syntax

Chinese differs from English in several ways in terms of the syntactic features. First, one critical feature of the Chinese language is that unlike English, which is defined as an inflectional language, there are few explicit syntactic or grammatical markers in Chinese (e.g., Lin, 2006; Tong and McBride, 2017). In an inflectional language like English, grammatical features such as tense and plural are indexed by inflections such as -ed, -ing, or -es. However, in Chinese, there are no explicit markers or inflectional indictors to mark the grammatical categories (e.g., Tong and McBride, 2017). For example, there is no difference in tense or number for the verb 请 (qing, “invite”) in the following two sentences (1) and (2).


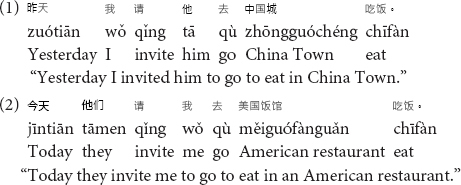


Moreover, the part of speech and meaning of a Chinese word is highly context-dependent in Chinese, more so than in English (e.g., Tong and McBride, 2017). For example, 合作 (hé zuò) could be a verb (to cooperate) or a noun (cooperation) depending on the contexts. In (3), 合作 (hé zuò) is used as a noun but in (4), it is used as a verb.


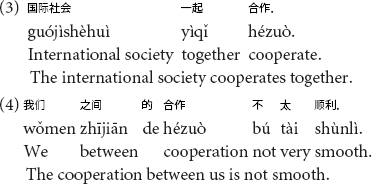


Since there is no inflectional system, Chinese readers rely on their syntactic knowledge to solicit information about the degree, tense, and parts of speech from linguistic constituents and their semantic relations (Lin, 2006). One of the most important types of syntactic knowledge is the word order knowledge. Different from English, Chinese has a more flexible word order (Zhao et al., 2021a). The basic word order in Chinese is SVO. For example, in 我爱吃鱼，我 (I) is the subject, 爱吃 (love to eat) are two verbs serving as the predicate, and 鱼(fish) is a noun serving as the object. Besides the typical subject-verb structure, there is also the topic-comment structure (Chao, 1968; Sun, 2006). The topic is about which something is said and may stand in several different logical relationships to the comment (Li and Thompson, 1989). In the above sentence, the object 鱼(fish) can be moved to the beginning of the sentence to form a topic-comment sentence (鱼，我爱吃), which means “As for fish, I love to eat (it).” In 饭吃完了(the food was finished), 饭(food) is the recipient of the action. In 大碗喝汤，小碗吃鱼(Big bowl, drink soup; small bowl, eat fish), the topics refer to the instruments of the verbs. Once a topic was established, it could be extended across succeeding sentences. For example, 鱼，我喜欢吃。做法也各有不同 (Fish, I love to eat (it). The ways of cooking (fish) also vary.). The flexibility of words in occurring at the beginnings, or ends, of Chinese sentences are due to some common syntactic properties (Sun, 2006, p.148). Given those specific features of Chinese sentence structures, some Chinese linguists argue that word order is one of the essential elements for readers to comprehend texts in Chinese (e.g., Li and Thompson, 1989) or even the most important syntactic device in Chinese (e.g., Chao, 1968). The syntactic knowledge of word order is important to trace the logic and semantic relations not only among words and phrases in a sentence but also across sentences, thereby contributing to passage-level comprehension (Tong et al., 2014; Zhao et al., 2021a). In L1 Chinese reading, children’s word order knowledge has been found to be a strong correlate of Chinese word recognition (e.g., So and Siegel, 1997) and reading comprehension (e.g., Yeung et al., 2013). Thus, it is logical to operationalize syntactic awareness as word order knowledge using a word order knowledge test. In addition to word order knowledge, previous research in L1 Chinese and L2 English reading has also shown that the ability to detect and correct grammatical errors accounted for a significant amount of unique variance in L1 Chinese (e.g., Chik et al., 2012) and L2 English reading comprehension (e.g., Li et al., 2021). Grammatical judgment and correction test could tap into different aspects of Chinese syntactic knowledge such as function words, conjunctions, tense markers, classifiers, particles, prepositions, and copula words. Thus, to further discern the syntactic skills important for reading comprehension in Chinese, the present study measured two types of syntactic awareness commonly assessed in L1 Chinese reading: word order knowledge (through a word order knowledge test) and morphosyntactic knowledge (through a grammatical judgment/correction test).

It needs to be noted that another type of test, conjunction cloze task, was also commonly used to measure syntactic awareness in L1Chinese reading research (e.g., Tong et al., 2014; Zhao et al., 2021a). This study did not adopt the conjunction cloze task mainly because although the knowledge about conjunctions can reflect syntactic knowledge, there are only a limited number of conjunctions in Chinese and most of them could be omitted in contexts (Sun, 2006). Thus, considering the content validity, this study adopted the word order knowledge test and the grammatical judgment/correction test to operationalize L2 Chinese syntactic awareness.

## The Purpose of This Study

As mentioned, current literature focuses on the role of syntactic awareness in L1 Chinese reading among native Chinese children. The role of syntactic awareness in L2 Chinese reading among adult CFL or CSL learners have yet to be established. Furthermore, it is unclear how different types of syntactic awareness are associated with how reading comprehension is measured. Therefore, the present study aimed at examining of the role of syntactic awareness in L2 Chinese reading. Previous studies in L2 Chinese reading (e.g., Zhou, 2018, 2021) indicated that character knowledge, vocabulary knowledge and morphological awareness contributed to L2 Chinese reading comprehension. The present study included linguistics skills such as character recognition, vocabulary knowledge, and morphological awareness to control for their effects over L2 Chinese reading comprehension. The present study also included some background variables such as age, major, gender, and length of learning Chinese. Although background variables were not commonly considered as predicting variables in regression analyses, this study included them to control for the effects of background variables to L2 Chinese reading comprehension. Categorial variables such as gender and major were dummy coded to be included in the regression analyses. The present study was interested in whether syntactic awareness will play a unique contribution to L2 Chinese reading comprehension beyond other linguistic skills and background variables. Furthermore, the present study aimed to investigate the relative contribution of two syntactic components (grammatical judgment/correction and word order) to two measures of L2 Chinese reading comprehension (multiple-choice questions test and cloze test). Two research questions were investigated.

1. What is the relative contribution of syntactic awareness to L2 Chinese reading comprehension among adult CFL learners?
2. How are the two tasks of syntactic awareness (the grammatical judgment/correction task and the word order task) contribute to the two measures of L2 Chinese reading comprehension (the multiple-choice questions test and the cloze test) differently?

## Materials and Methods

### Participants

The participants were 209 adult CSL learners studying abroad in four Chinese universities located in Beijing city and Gansu province. The mean age of the participants was 21.53 (*SD* = 7.97). The participants had averagely learned Chinese for 31.24 months, *SD* = 16.06. The majority of the participants’ majors were related to Chinese language or culture (e.g., 50% in teaching Chinese as a foreign/second language; 28.1% in Chinese linguistics, and 2.3% in Chinese studies). 99 of the participants (47.4%) were male and 109 (52.4%) were female. Based on self-reported proficiency and proficiency test (HSK) scores, the proficiency level of the participants was intermediate to advanced.

### Measures

A character knowledge test, a vocabulary knowledge test, a morphological awareness test, a word order test, a grammatical judgment/correction test, and two reading comprehension tests were administered to the participants. Please see the Appendix for all the tests.

#### Character Knowledge Test

A character knowledge test adopted from Zhou (2018) was used to measure learners’ receptive knowledge of Chinese characters. There were 30 real characters, nine pseudo-characters, and six easy characters which were simple in form and presumed well known by the participants. The test randomly chose the real characters from the character frequency list (10 from beginning, intermediate, and advanced lists, respectively) in *The Graded Chinese Syllables, Characters, and Words for the Application of Teaching Chinese to the Speakers of Other Languages* (Liu and Ma, 2010). Nine pseudo-characters were created to control for guessing. No participants in this study indicated that they knew a pseudo-character. Six easy characters such as 一，人，山 were added as fillers.

#### Vocabulary Knowledge Test

This test was adopted from Zhou (2018). The format of the test was based on Guo and Roehrig (2011); Nation (2001), and Shiotsu and Weir (2007). The test randomly selected thirty words from the beginning, intermediate, and advanced word frequency lists from *The Graded Chinese Syllables, Characters, and Words for the Application of Teaching Chinese to the Speakers of Other languages* (Liu and Ma, 2010). Another 30 words were added as distractors. The test adopted a matching format. Three target words and three distractors were placed in one block. The participants were asked to select the word on the left that matches the explanation on the right.

#### Morphological Awareness

Adopted from Zhou (2018), this test aimed to investigate participants’ ability to distinguish compound structures in Chinese. Compounding is the main way to form words in Chinese (Sun, 2006). Five types of syntactic structures in Chinese disyllabic words were covered in the test: juxtapositional, modificational, governmental, predicational, and complemental (please see Yip, 2000 for the five types and examples). The participants were asked to choose the word whose morphemes went together in a similar way with the target word. For example, the compound structure of the target word “喝水” (hēshuĭ, to drink+water = to drink water) was governmental (i.e., verb+object). Thus, among three options “睡觉” (shuìjiào, to sleep+sleep = to have a sleep, governmental), 出去 (chūqù, exit+out = go out, complemental) and 肥胖 (féi pàng, fat+fat = obesity, juxtopositional), “睡觉” shared the same compound structure with “喝水.”

#### Word Order Test

This test was based on Nassaji (2003) and Yeung et al. (2013). Seven simple and eight complex sentences were prepared. Each sentence was divided into six to eight segments and scrambled. The participants were asked to rearrange the segments to form meaningful and syntactically correct sentences. Each correct arranged sentence was credited with two points. Some sentences had multiple correct word orders, which were all marked correctly. As for grading, if half of the sentence was correctly rearranged, one point was given. 0.5 point was credited if two continuously segments were correctly arranged. For example, for the item 6 ①环境 ②越来越好 ③城市的 ④变得 ⑤这个 ⑥了. If the answer was ⑤这个③城市的①环境④变得②越来越好⑥了, two points were given. If the answer was ⑤这个③城市的①环境⑥了②越来越好④变得, since the first half of the sentence was correct (⑤③①), but the second half was not correctly rearranged (⑥②④ instead of ④②⑥), one point was credited. If the correct answer was ⑤这个③城市的 ⑥了①环境②越来越好④变得, since only two continuously segments were correctly rearranged (⑤③), 0.5 point was credited.

#### Grammaticality Judgment/Correction Test

This test was adopted from Zhou (2018) and the original test was based on Tong et al. (2014) and Yeung et al. (2013). There were sixteen syntactically incorrect sentences, which were designed to test different aspects of syntactic knowledge such as function words, conjunctions, tense markers, classifiers, particles, prepositions, and copula words. The participants were asked to identify the mistakes and make corrections.

#### Multiple-Choice Questions Test

This test was adopted from Zhou (2018). Four reading passages were selected from Chinese books or websites. The lengths of the passages were 207, 337, 359, and 595 characters, respectively. Passage 1 and Passage 3 were narratives, Passage 2 was a fable selected from a graded reader, and Passage 4 was a persuasive article chosen from an online version of a Chinese newspaper. Passage 1 and 2 were modified and difficult words were replaced with easier ones. Passage 3 and 4 were authentic texts, with no changes made to the texts. To determine whether the readings were at the appropriate levels for the participants of this study, Chinese Readability Index Explorer for Chinese as a Foreign Language (CRIE-CFL) (Sung et al., 2015) was used to check the readability levels of the four reading passages. The results showed that Passage 1 and 2 were at B1, Passage 3 at B2, and Passage 4 at C2 levels. The difficulty levels of the four passages were ideal for the participants in this study.

#### Cloze Test

Cloze tests are usually constructed by deleting every n-th word from a passage and ask the test takers to restore the deleted words (e.g., Alderson, 2000, p. 207). The cloze test was adopted from Zhou (2018). There were altogether two passages. The first sentence of each passage remained intact to provide some contextual support. Starting from the second sentence, every 6th Chinese character was deleted. The participants were asked to write down the missing character. If the participants did not know how to write the characters, they were instructed to write down pinyins with correct tones. Each correct character or pinyin with tone was credited with one point.

### Procedures

All the tests were administered in pencil and paper format in classrooms. The participants were explained to the research purpose and signed the consent forms. They were asked to fill in the background information form first where the information on age, gender, major, and length of learning Chinese was obtained. The tests were then administered in groups. The duration of the test session was around 90 min. Tests were administered in the following order: the character knowledge test, the vocabulary knowledge test, the morphological awareness test, the grammatical judgment/correction test, the word order test, the multiple choice questions, and the cloze test.

### Data Analysis

The data of this study were first checked to see whether the assumptions to conduct hierarchical regression analyses were met. The descriptive statistics and correlations of all the variables were reported. To answer RQ1, the raw scores of the two measures of reading comprehension, namely the multiple-choice questions test and the cloze test, were added to form a total score of L2 Chinese reading comprehension (i.e., raw score total). The z-scores of each test were also saved and combined to form a reading comprehension total score (i.e., z-score total). Followed-up regression analyses showed that raw score total and z-score total did not make a difference, thus this study used the reading comprehension raw score total. To further explore how the two types of syntactic awareness tasks might associate with the multiple-choice questions test and the cloze test differently, the multiple-choice questions and the cloze test were treated as dependent variables separately. Another two hierarchical regression analyses were run to answer RQ2.

## Results

### Descriptive Statistics

Table 1 presents the means, standard deviations, number of items in each test, and reliability coefficients computed for the various tasks in this study. All the reliability coefficients were in high range (0.783 to 0.890).

**TABLE 1:** Descriptive Statistics of all the Variables.

Variables	M	SD	Reliability	# Items
Age	21.53	7.97		
Length of learning Chinese (Months)	31.24	16.06		
Character knowledge	20.56	6.59	0.881	30
Vocabulary knowledge	16.51	6.83	0.816	30
Morphological awareness	6.97	3.76	0.789	15
Grammatical judgment/correction test	14.72	8.66	0.813	15
Word order test	18.15	5.59	0.814	15
RC-Multiple-choice questions test	22.26	8.06	0.783	20
RC-Cloze test	12.78	8.94	0.890	37
RC-Total	34.98	15.37	0.903	

*RC, reading comprehension.*

#### Correlation

Table 2 shows the correlations among all the measures in the present study. Age was significantly positively correlated with vocabulary knowledge, character knowledge, and reading comprehension (total), and multiple-choice questions, but not with other variables. Length of learning Chinese was positively correlated with all the component skills and two measures of reading comprehension (*rs* > 0.207, *p* = 0.01). The character knowledge, the vocabulary knowledge, the morphological awareness, the grammatical judgment/correction test, and the word order were all significantly positively correlated with the multiple-choice questions test and the cloze test (*r* ranging from 0.424 to 0.739, *p* = 0.01). The character knowledge, the vocabulary knowledge, the morphological awareness, and the two measures of syntactic awareness were also all positively correlated with each other (*rs* > 0.377, *p* = 0.01). The partial correlation underscored the importance of syntactic awareness as well as other linguistic skills in explaining L2 Chinese adult readers’ performance in passage-level reading comprehension.

**TABLE 2:** Partial correlation among all variables.

Variables	1	2	3	4	5	6	7	8	9	10
1. Age	1									
2. Length of LC (months)	−0.037	1								
3. Character knowledge	0.167*	0.223**	1							
4. Vocabulary knowledge	0.197**	0.357**	0.616**	1						
5. Morphological awareness	0.101	0.207**	0.433**	0.573**	1					
6. Grammatical judgment/correction test	0.116	0.322**	0.377**	0.621**	0.419**	1				
7. Word order test	0.122	0.306**	0.486**	0.727**	0.438**	0.669**	1			
8. RC-Multiple-choice questions test	0.225**	0.298**	0.471**	0.739**	0.446**	0.556**	0.682**	1		
9. RC-Cloze test	0.109	0.230**	0.437**	0.698**	0.424**	0.624**	0.668**	0.639**		
10. RC (Total)	0.180*	0.263**	0.497**	0.777**	0.465**	0.643**	0.742**	0.896 **	0.915**	1

LC, learning Chinese; RC, reading comprehension. *p < 0.05; **p < 0.01.

### Hierarchical Multiple Regression

A series of hierarchical multiple regression analyses were conducted. In the first hierarchical regression analysis, reading comprehension total was the dependent variable. To control for the effects of background variables to linguistic-related variables, background variables (age, major, gender, and length of learning Chinese) were first entered into the equation. Next, the linguistic variables (the character knowledge, the vocabulary knowledge, and the morphological awareness) were entered into the equation. All of those linguistic variables have been found to be associated with reading comprehension in previous studies (e.g., Zhou, 2018). Two measures of syntactic awareness: the grammatical judgment/correction test and the word order test, were entered in the equation in the last step. Table 3 shows the results of the regression analyses. Age, gender, major, and length of learning Chinese explained 31.9% of the variance in reading comprehension total. Character knowledge, vocabulary knowledge, and morphological awareness added an extra 31.3% of the variance to reading comprehension total. After controlling for the effects of background related variables and the character knowledge, the vocabulary knowledge, and the morphological awareness, the syntactic awareness made a unique contribution to L2 Chinese reading comprehension total, explaining 6.9% of the variance in reading comprehension total. Totally, the predicting variables explained 70% of the variance in L2 Chinese reading comprehension total.

**TABLE 3:** Multiple regression analysis predicting passage-level reading comprehension.

Step	Variables	β	*T*	*R* ^2^	Δ*R*^2^
Step 1	Age	0.198	0.885	0.319	0.319
	Gender	0.906	0.646		
	Major	0.366	2.921**		
	Length of learning Chinese	−0.003	−0.065		
Step 2	Character knowledge	0.053	0.422	0.632	0.313***
	Morphological awareness	−0.008	−0.036		
	Vocabulary knowledge	0.942	5.544***		
Step 3	Word order	0.757	4.129**	0.7	0.069**
	Grammatical judgment/correction test	0.302	2.882***		

**p < 0.01 and ***p < 0.001.

In addition, this study separately analyzed the associations between the two types of reading comprehension tests, that is the multiple-choice questions and the cloze test, and the syntactic awareness tasks, that is the grammatical judgment/correct test and the word order test. The results are presented in Table 4. Table 4 shows that the grammatical judgment/correction test and the word order test together contributed 3.2% of unique variance to the multiple-choice questions and 8.9% to the cloze test even after controlling for the contributions of age, major, gender, length of learning Chinese, character knowledge, vocabulary knowledge, and morphological awareness. Both the grammatical judgment test and the word order test were significant predictors of the cloze test. However, only word order was a significant predictor to the multiple-choice questions test. Thus, it seems that word order knowledge had a stronger predicting power to L2 Chinese reading comprehension compared to grammatical judgment/correction ability.

**TABLE 4:** Multiple regression analysis predicting two types of reading comprehension questions.

Step	Variables	β	*t*	*R* ^2^	Δ*R*^2^
Dependent variable	Multiple-choice questions				
Step 1	Age	0.283	2.117*	0.318	0.318
	Gender	1.416	1.69		
	Major	0.15	2.016*		
	Length of learning Chinese	0.022	0.85		
Step 2	Character recognition	−0.007	−0.094	0.57	0.252***
	Morphological awareness	0.05	0.409		
	Vocabulary knowledge	0.508	5.024***		
Step 3	Word order	0.349	3.175**	0.602	0.032**
	Grammatical judgment/correction test	0.043	0.693		
Dependent variable	Cloze test				
Step 1	Age	−0.085	−0.548	0.22	0.22
	Gender	−0.543	−0.557		
	Major	0.211	2.427*		
	Length of LC	−0.023	−0.792		
Step 2	Character recognition	0.063	0.712	0.464	0.265***
	Morphological awareness	−0.067	−0.465		
	Vocabulary knowledge	0.439	3.718***		
Step 3	Word order	0.408	3.201**	0.551	0.089***
	Grammatical judgment/correction test	0.258	3.542**		

*p < 0.05; **p < 0.01; ***p < 0.001.

## Discussion

To reiterate, the aims of the present study are (1) to examine the unique contribution of syntactic awareness to L2 Chinese reading comprehension among adult CSL learners beyond a number of linguistic and background variables; and (2) to show the relative significance of two syntactic awareness tasks (the grammatical judgment/correction task and the word order task) in two measures of L2 Chinese reading comprehension (the multiple-choice questions test and the cloze test). The hierarchical regression analyses results showed that syntactic awareness uniquely contributed to L2 Chinese passage-level reading comprehension by explaining 6.9% of its variance. The findings suggested that the strong correlation between syntactic awareness and discourse-level reading comprehension was beyond the contribution of character knowledge, vocabulary knowledge, morphological awareness, and background variables such as age, major, gender, and length of learning Chinese. Those results were in line with previous work examining the associations of syntactic awareness and reading comprehension in alphabetic languages (e.g., Muter et al., 2004; Nation and Snowling, 2004) and in L1 Chinese (e.g., Yeung et al., 2013; Tong et al. 2014; Zhao et al., 2021b).

The findings of this study showed that the correlation between the grammatical judgment/correction and the L2 Chinese reading comprehension total was 0.643, and between the word order test and the L2 Chinese reading comprehension was 0.742. Existing research have shown that syntactic awareness correlates with reading difficulties or reading comprehension among readers with disabilities and fluent readers (e.g., Geva and Massey-Garrison, 2013; Farnia and Geva, 2019; Li et al., 2021). Jeon and Yamashita’s (2014) meta-analysis showed that L2 syntactic knowledge, L2 vocabulary knowledge, and L2 decoding were the three strongest correlates of L2 reading comprehension, with grammatical knowledge being the strongest among the three (correlation of 0.85 compared to 0.79 and 0.56). Similarly, van Gelderen et al. (2004) reported a correlation of 0.80 between L2 grammar knowledge and reading. This study extended this line of research to L2 Chinese reading.

The significant role that the syntactic awareness played in L2 Chinese reading comprehension was not surprising. There are at least two potential explanations for the associations between syntactic awareness and reading comprehension. First, syntactic awareness facilitates the reading comprehension. Syntactic information from determiners (this, that), word order, subordinate clauses, conjunctions, prepositions, tense, among other information, provides ongoing instructions for the construction of meaning (e.g., Grabe, 2009). The process of parsing incoming structural information that supports comprehension is happening every second during fluent reading. As a reader begins to look at a text, word recognition processes begin. The first words are recognized, and the extract of syntactic information also begins (e.g., Grabe, 2009). For example, the measure word 个 in Chinese signals that a noun will follow; 虽然 (although) signals that there will be a transition in meaning and it is highly possible that readers will see 但是 (but) in the following clause. Thus, a good mastery of syntactic knowledge facilitates the comprehension process and speeds up the comprehension. Secondly, syntactic awareness facilitates the comprehension monitoring. Reading comprehension is a process that requires comprehension monitoring (e.g., Cain et al., 2004), which refers to the process of “keeping track of the success with which one’s comprehension is proceeding, ensuring that the process continues smoothly, and taking remedial action if necessary” (Baker and Brown, 1984, p. 355). It is not uncommon for readers to find that comprehension may fail or become severely constrained while reading. This lack of comprehension may be driven by a few unknown key words, by an inability to sort out semantic relations between nouns and verbs in complex sentences, or by syntactic complexity of some long sentences (e.g., Grabe, 2009). Syntactic knowledge may help readers monitor their comprehension more effectively (Tunmer et al., 1987). Research has shown that skilled readers with good sense of syntactic awareness are able to recognize the sense of the lack of comprehension more easily and took measures to address the problem. However, unskilled readers with poor syntactic awareness were unable to detect the lack of comprehension and/or employ repair strategies that were essential to improve the understanding of a text (e.g., Tunmer et al., 1987; Grabe, 2009; Tong et al., 2014).

Besides extending the significant role of syntactic awareness to L2 Chinese reading comprehension, this study also examined the associations between the types of syntactic awareness and the measures of reading comprehension. Theoretically, it is interesting to examine whether the associations of syntactic awareness with reading comprehension differed across the two different reading comprehension tasks: the multiple-choice questions test and the cloze test. In doing this, this study conducted two sets of regression analyses by entering scores for the multiple-choice questions test and the cloze test as dependent variables, respectively. The two syntactic tasks explained 3.2% of the total variance in the multiple-choice questions test and 8.9% in the cloze test. Given that the cloze test was usually designed to measure local understanding while the multiple-choice questions test was more often designed to test global understanding (e.g., Alderson, 2000), the findings of this study suggested that the word order knowledge may be important to both the local and global understanding of Chinese passages while the grammatical judgment/correction ability may only play an important role in the local understanding of L2 Chinese reading passages. Thus, methodologically, it is worth pointing out that the cloze test and the grammatical judgment/correction test both require a process of searching and matching (e.g., Weaver and Wendell, 1965). Alderson (2000) stated that a cloze test is word-based, and many cloze items in a cloze test are constrained by the immediate sentence constituents and may not by long range discourse. Similarly, the grammatical judgment/correction test requires the participants to identify and correct grammar errors at sentence levels. Given the similarity in the testing formats, it has been suggested that the performance on those types of tests correlates highly (e.g., Kennedy and Weener, 1973; Tong et al., 2014). In this study, the grammatical judgment/correction test is correlated with the cloze test at *r* = 0.624, higher than *r* = 0.556 between the grammatical judgment/correction test and the multiple-choice questions test. This is an important finding, and future research may continue this line of research and examine how other types of syntactic awareness and ways of measuring reading comprehension are associated.

The present finding that syntactic awareness uniquely predicted L2 Chinese passage level reading comprehension among adult learners is of both theoretical and practical importance. From a theoretical perspective, the present study, for the first time, highlighted the importance of syntactic awareness as an important component of L2 Chinese reading beyond age, major, gender, length of learning Chinese, character knowledge, vocabulary knowledge, and morphological awareness. From a practical perspective, the finding that syntactic awareness was significantly predictive of L2 adult leaners’ reading comprehension is of particular interest for L2 Chinese teachers. Syntactic skills should be taught and practiced along with the teaching of other component skills such as vocabulary knowledge and morphological awareness to reduce L2 Chinese reading anxiety and improve L2 Chinese reading fluency and comprehension (e.g., Zhou, 2017; Zhou and Day, 2020).

Future work might attempt to replicate and extend the findings of this paper by examining the effects of other syntactic skills, for example, the conjunction pair knowledge, to L2 Chinese reading. Moreover, the role of syntactic awareness to L2 Chinese reading among readers of different reading abilities is also worth exploration.

## Data Availability Statement

The raw data supporting the conclusion of this article will be made available by the authors, without undue reservation.

## Ethics Statement

The studies involving human participants were reviewed and approved by University of Hawaii at Manoa. The patients/participants provided their written informed consent to participate in this study.

## Author Contributions

The author confirms being the sole contributor of this work and has approved it for publication.

## Conflict of Interest

The author declares that the research was conducted in the absence of any commercial or financial relationships that could be construed as a potential conflict of interest.

## Publisher’s Note

All claims expressed in this article are solely those of the authors and do not necessarily represent those of their affiliated organizations, or those of the publisher, the editors and the reviewers. Any product that may be evaluated in this article, or claim that may be made by its manufacturer, is not guaranteed or endorsed by the publisher.
