# Supplementary material for: The Effects of Syntactic Awareness to L2 Chinese Passage-Level Reading Comprehension
Source: Front Psychol. 2022 Feb 1;12:783827. doi: 10.3389/fpsyg.2021.783827 (PMC8844565; doi:10.3389/fpsyg.2021.783827)
Supplement: Supplementary file 1 [file Image_1.pdf]

Appendix

The character knowledge Test

Instruction: please decide ***whether you know*** the following characters. If you know them, please put Y after the characters. If not, please put N after the characters.

请判断您是否认识下面的这些汉字，如果认识，请写 Y， 如果不认识，请写 N。

	Y/N		Y/N		Y/N
爱		蹦		笔	
丑		王		狗	
小		[illegible]		子	
癌		包		钉	
菜		泪		蜂	
姑		脏		倦	
[illegible]		大		踷	
人		伊		一	
禽		云		军	
紫		破		花	
[illegible]		罪		舟	
水		明		倚	
爹		火		鼠	
桥		寺		浊	
钌		戴		节	

The vocabulary knowledge test

Instruction: Please choose the word on the left that matches the explanation of the word on the right.

请从左边选出和右边解释相匹配的词。

e.g.	

1.老师<br>2.树木<br>3.杂志<br>4.花朵<br>5.跑步<br>6.垃圾	（ 1 ）工作是教学生<br>（ 3 ）可以阅读的<br>（ 5 ）一种锻炼身体的方式
1. 出院<br>2. 饮料<br>3. 房屋<br>4. 忽然<br>5. 上课<br>6. 财产	（ ）住的地方<br>（ ）病好了离开<br>（ ）出乎意料
1. 书本<br>2. 出发<br>3. 买菜<br>4. 举手<br>5. 名称<br>6.人工	（ ）王先生<br>（ ）不是天然的<br>（ ）问问题前会做这个动作
1. 吃惊<br>2. 特色<br>3. 小时候<br>4. 放学<br>5. 咱们<br>6. 回家	（ ）与众不同的方面<br>（ ）还没有长大<br>（ ）包括你和我
1. 做法<br>2. 成语<br>3. 做工<br>4. 独自<br>5. 说话<br>6. 群众	（ ）做事情的方式<br>（ ）一种固定的表达方式<br>（ ）一个人

1. 咖啡<br>2. 过度<br>3. 聚会<br>4. 名胜<br>5. 初中<br>6. 竞赛	（　　）风景很漂亮的地方<br>（　　）超出可接受的水平<br>（　　）可以判断谁在某一方面更强
1. 前进<br>2. 诋毁<br>3. 认同<br>4. 特性<br>5. 欣赏<br>6. 寿司	（　　）和其他事物不同的品质<br>（　　）赞成别人的做法或价值观<br>（　　）特别喜欢崇拜某一个人
1.乐曲<br>2.做客<br>3.参加<br>4. 出格<br>5. 合适<br>6. 比赛	（　　）弹钢琴会演奏出的东西<br>（　　）去别人家里<br>（　　）比较恰当
1. 残害<br>2. 起草<br>3. 健身<br>4. 口径<br>5. 何时<br>6. 粉丝	（　　）拟定初稿<br>（　　）处理问题的原则<br>（　　）一个疑问词
1. 缩水<br>2.地板<br>3. 演技<br>4. 演出<br>5. 建设<br>6.坐落	（　　）有的衣服洗了之后会<br>（　　）指演员的专业水平<br>（　　）指建筑物的位置

1. 焕发<br>2. 铸造<br>3. 水涨船高<br>4. 散户<br>5. 买卖<br>6. 作对	（　　　）形容人的精神状态好<br>（　　　）比喻一个事物随着另一事物变化而变化<br>（　　　） 不合作

The morphological awareness test

Instruction: please choose the word whose characters go together in a similar way to the target word.

请选出和目标词结构相似的词。

Target word	A	b	c	Answer
e. g. 喝水	a. 出去	b. 睡觉	c. 肥胖	b
美丽	a. 艰难	b. 打死	c. 青山	
长城	a. 爱好	b. 安静	c. 春天	
出国	a. 答应	b. 发言	c. 根本	
感到	a. 公路	b. 吃完	c. 关心	
快餐	a. 留下	b. 篮球	c. 流行	
记住	a. 坚强	b. 做好	c. 家人	
破坏	a. 骑车	b. 气温	c. 剪断	
食物	a. 设计	b. 收费	c. 玩具	
头脑	a. 校长	b. 痛苦	c. 晚安	
重点	a. 英语	b. 资金	c. 转变	
走开	a. 做到	b. 座位	c. 今天	
门票	a. 领导	b. 名单	c. 离开	
交费	a. 京剧	b. 开会	c. 解开	

继续	a. 加油	b. 检查	c. 画家	
初级	a. 变成	b. 吃饭	c. 菜单	

The grammatical judgment/correction test

Instruction: There is one grammatical error in each sentence. Please underline the ungrammatical part and provide a correction. You can delete the error part, replace it with a different word/character, or move its location.

下面每个句子都有一个语法错误。请在错误的部分下面划线并改正。你可以删除错误部分，用其他词/字替换，或改变错误部分的位置。

E.g. 除了小王以外，我们班的同学还会说日语。
Correction: change 还 to 都
1.我是 2015 年去中国了。
Correction:
2. 已经 11 点了，我估计他今天一定不会来参观画展了。
Correction:
3. 难道你连这个规定还不知道吗？
Correction:
4. 你吃饭过没有？
Correction:
5. 你什么没去看电影？
Correction:
6. 这个客厅大是大，不过坐得下十个人。
Correction:
7. 我的面包把狗吃了。
Correction:
8. 我的姐姐比你的姐姐很美。
Correction:
9. 爸爸花了一个小时把早饭准备成了。
Correction:
10. 她笑了对我说,“我爱你！”
Correction:
11. 给我打电话还是发短信都可以。
Correction:

12. 昨天我见了我的女朋友，今天我再要跟她见面。
Correction:
13. 小美哭得两双眼睛都变红了。
Correction:
14. 丽丽的男朋友是很帅。
Correction:
15. 你做的很对，不要在乎别人怎么想。
Correction:
16. 学校向我家很近。
Correction:

The word order test

Instruction: please put the following segments in order to form a sentence. You can add punctuation when it is necessary.

请把下面的部分排序，组成一个句子。您可以在需要的时候添加标点符号。

E.g. ①我 ②心情 ③好 ④非常 ⑤ 今天 。
Answer: ① ⑤ ② ④ ③
1. ①桌子 ②放着 ③书 ④上 ⑤ 一本
Answer:
2. ①地方 ②很多 ③云南 ④在 ⑤玩了 ⑥他
Answer:
3. ①对 ② 小王 ③兴趣 ④工作 ⑤ 自己的 ⑥没
Answer:
4. ①就 ②马上 ③了 ④要 ⑤超市 ⑥关门
Answer:
5. ①刚才 ②很冷 ③明天 ④说 ⑤电视里 ⑥天气
Answer:
6. ①环境 ②越来越好 ③城市的 ④变得 ⑤这个 ⑥了
Answer:
7. ①那 ②不 ③衣服 ④ 他的 ⑤是 ⑥件
Answer:
8. ①怎么变 ②其实是 ③寻找快乐的心 ④不管 ⑤大环境 ⑥一种习惯
Answer:
9. ①再检查 ②看 ③你 ④ 有没有 ⑤ 最好 ⑥还 ⑦一下 ⑧问题

Answer:
10. ①很快 ②女朋友 ③恢复了④照顾下 ⑤他 ⑥在 ⑦健康 ⑧的
Answer:
11. ①都有 ②幸福 ③的 ④理解 ⑤含义 ⑥对于 ⑦不同的 ⑧每个人
Answer:
12. ①几个朋友 ②开车 ③周末 ④都不会 ⑤我的 ⑥想去郊游 ⑦他们 ⑧但是
Answer:
13. ①在使用上 ②进餐工具 ③最主要的 ④中餐 ⑤筷子是 ⑥讲究 ⑦很多 ⑧ 也有
Answer:
14. ①都应该 ②成功 ③获得 ④失败 ⑤努力过的人 ⑥无论 ⑦还是 ⑧鲜花和掌声
Answer:
15. ①中国的 ②代替父亲 ③女英雄 ④ 而闻名天下 ⑤花木兰是 ⑥以 ⑦并打败入侵敌人 ⑧参加军队
Answer:

The multiple choice-questions test

Instruction: please read the following passages and answer the questions.
请阅读下面的文章，并回答问题。

Passage 1

我的左邻过去是一位歌星，天天躲在房子里听她自己当年演唱的唱片。右邻是一位退休的教授，天天喃喃地祈祷着什么。在我的想象中，这位教授一定很老了。

事实却不是这样的，我发现 70 岁的教授精神很好，走路时步子轻快，眼睛里闪着喜悦的光。可是那位歌星，40 多岁就已经精神很不好，腿脚也不灵便了。

原来这位歌星天天回忆过去美好的日子，对现在的生活很不满意 。而老教授虽然不再教书了，退休后却又开始学习拉丁文。他说："每多认识一个生字，我就觉得年轻了一岁。"我听到的"祈祷"声，其实就是他的读书声。

According to the above passage, please mark the statements 1-5 with (**T**) if it is true or (**F**) if it is false.
根据上面的文章，请判断下面的句子是对(T)还是错(F).

1. 教授的年龄比歌星大。(　　　　　)

2. 歌星的精神比教授好。 (　　　　　)

3. 这位歌星不喜欢自己现在的生活。(　　　　　)

4. 教授每天很忙，因为他要教学生。(　　　　　)

5. 教授身体好是因为他每天祈祷。(　　　　　)

Passage 2

从前，在一座很大的森林里住着很多动物。森林里有一只老虎，他是这座森林里最强壮的动物，所有的动物都害怕它，一看到老虎来了，就都跑得远远的，这只老虎非常得意，它经常在森林里走来走去，觉得自己真了不起。

有一天，老虎正在森林走着，忽然一只狐狸从树丛里跳了出来，跳到老虎面前。原来这只狐狸没有看到老虎。老虎很生气，它一把捉住这只大胆的狐狸，要把狐狸吃了。

狡猾的狐狸看到自己已经不能从老虎手里逃走了，就想出了一个办法。它对老虎说：“你不能吃我！”老虎愣住了，就问：“为什么？”狐狸说：“玉皇大帝（Jade Emperor）派我来当这个森林的大王，谁敢吃我? 如果你不相信，我们一起在森林里走一走，看看动物们怕不怕我？”

老虎同意了。他让狐狸走到前面，自己跟在后面。 动物们看到老虎跟在狐狸的后面，都吓得赶快逃走了。这时，狐狸得意地对老虎说：“看到了吗？动物们都怕我呢！”老虎也认为动物们现在怕的是狐狸，只好把狐狸放走了。

6.下面哪项是正确的 (　　　　　)

A 老虎是最强壮的动物之一　　B 动物们都喜欢跑步

C 老虎喜欢跑来跑去　　D 动物们害怕老虎

7. 狐狸跳到老虎前面是因为：(　　　　　)

A 它想吃老虎　　B 它想吓老虎

C 它没看见老虎　　D 它对老虎很生气

8. 第三段的“愣住”最可能的意思是：(　　　　　)

A 笑　　B 伤心　　C 生气　　D 呆

9. 老虎放了狐狸是因为: (　　　　　)

A 狐狸是森林的大王　　　　**B 动物们都怕狐狸**

C 老虎怕玉皇大帝　　　　**D 老虎以为动物怕狐狸**

10. 根据上文，可以知道老虎：(　　　　　)

A 很胆小　　**B 很友好**　　**C 很糊涂**　　**D 很善良**

Passage 3

记得十年前一个寒冷的冬天，我住在屏东市一家满是臭虫的旅店。为了看内埔乡稻田的日出，我凌晨四点就从旅店出发，赶到内埔乡时天色还是昏暗的，我就躺在田埂边的草地上等候，没想竟昏沉沉地睡去了，醒来的时候日头已近中天。

我捶胸顿足，想到走了一个小时的夜路，难过得眼泪差一点落下来。正在这时，我看到田中的秧苗反射着阳光，田地因干旱而显出的裂纹，连绵到天边，有非常之美，是我从未见过的景象。我立即转悲为喜，感觉到如果能不执着，心境就会美好得多。

这时，一位农夫走来，好意地请我喝水，当他知道我是来看日出的美景时，抬头望着天空出神地说："如果能下雨，就比日出更美了。"我问他下雨有什么美，他说："这里闹干旱已经两个月了，没有下过一滴雨——日出有什么好呢？"我听了心里一惊，非常惭愧，以一种悔罪的心情看着天空的烈日，很能感受到农夫的忧伤。

后来，我和农夫一起向天空祈求下雨，我深切地感悟到：离开真实的生活，世间一切的美都显得虚幻不实。

11. "我"去屏东市干什么? (　　　　　)

A 去看田中的秧苗　　　　B 去稻田看日出

C 去看农夫朋友　　　　D 去祈求天空下雨

12. "我"是什么时候醒来的？(　　　　　)

A 凌晨四点钟　　　　B 快中午的时候

C 凌晨五点钟　　　　D 一小时以后

13. "我"为什么"捶胸顿足"？(　　　　　)

A 走路走得太久了　　　　B 走夜路走得太累了

C 睡过头了　　　　D 太难过了

14. 下列哪项说法正确？(　　　　　)

A 农夫不知道我来做什么　　B 我非常想喝水

C 稻田里很久没下雨了　　D 我和农夫都喜欢日出

15. 这个故事主要告诉我们什么？(　　　　)

A 人不能太执着　　B 下雨比日出更美

C 要多感受别人的悲伤　　D 美离不开真实的生活

Passage 4

相关研究显示，由于我国农村地区的青少年儿童食用越来越多高糖和高热量食品，肥胖比例呈快速增长。肥胖问题已非城里人和成年人的“专利”，农村青少年儿童正面临肥胖的侵袭。不同于城市，农村的儿童肥胖，有其独特的原因。

首先，随着农民收入水平提高，农村的饮食结构发生了变化。传统的清淡饮食开始向高脂肪、高热量、低纤维方向转变。“在农村，一些人觉得多吃油、多吃肉对身体好，有营养。”刘璐说，“此外，热量高的糕点和含糖饮料，也是诱发农村儿童肥胖的重要原因”。

记者调查发现，甜饮料、糕点在农村家庭很常见，成了孩子的主要零食。与此同时，在不少农村小卖部，“奥利奥”变成了“奥和奥”，“营养快线”变成了“营养干线”。一些专家表示，用料低劣、着色剂滥用、添加剂超标的假冒伪劣食品，也是造成农村儿童肥胖的重要原因。

其次，记者调查发现，农村健康营养知识匮乏。中国学生营养与健康促进会发布的《中国儿童少年营养与健康报告》显示，多数人不知道什么食物真正有营养，把鸡蛋卖了换方便面, 零食，用胡萝卜喂家禽。

“家长投其所好，孩子爱吃什么就买什么。许多家长以孩子不喜欢吃为由，放弃了培养孩子吃蔬菜、水果的饮食习惯，而选择了含糖饮料、油炸食品。”河南平舆县一名乡村教师说。

再次，受“小孩胖点好”的农村传统观念影响。“多吃一碗饭，多穿一尺布”是大部分农村老人对于肥胖的理解。加上追食、诱食、逼食等错误普遍存在的喂养方式，导致农村胖墩也越来越多。

“不少农村老人认为把孩子养得胖乎乎的是件好事，完全不知道胖是疾病的前期症状。”河南省肿瘤医院医生庄昊说，儿童肥胖如果不能及时控制和改善，严重的会得糖尿病、代谢紊乱等疾病。

16.农村人拿胡萝卜喂家禽说明了什么？(　　　　)

A 胡萝卜种得太多　　B 胡萝卜不好吃

C 胡萝卜没有营养　　D 农村人缺少营养知识

17. “多吃一碗饭，多穿一尺布”表明了农村老人的什么态度？(　　　　)

A 吃得多的话衣服也要穿大号的　　B 吃得多一点没有什么关系

C 吃得多的话孩子穿衣服不好看　　D 吃得多的话养育孩子很艰难

18.下面哪种食品最有可能存在质量问题？(　　　　　)

A 奥利奥　　　　B 营养干线　　　　C 甜饮料　　　　D 糕点

19.第四段的匮乏最有可能的意思是什么? (　　　　　)

A 丰富　　　　B 昂贵　　　　C 不足　　　　D 错误

20.上文主要介绍了：(　　　　　)

A 导致肥胖的食品泛滥　　　　B 肥胖问题的严重后果

C 农村儿童肥胖的原因　　　　D 农村的不良生活习惯

The cloze test

Instruction: Please fill in the following blanks with ***only one Chinese character***. If you don't know how to write the character, please write pinyin with tones.

请在每个空格中***只填入一个汉字***。如果你不会写汉字，请写拼音和声调。

Passage 1

森林里，动物们决定举办一个晚会，这次演出吸引了几乎所有的动物。他们都很积极，____备的节目各有_____点，小鸟要给大____唱歌，老虎要跳____，小猫要画画儿，____羊要讲故事，狮____说他给大家照____，熊猫说："我不会____演，但是我可以____观众，为大家鼓____。"最后只剩下小____了，她想了好久，_____然得意地说："我____责为大家送免____的牛奶！"

Passage 2

一个年轻人获得一份销售工作，勤勤恳恳干了大半年，却接连失败。而他的同事，个____都干出了成绩。____实在忍受不了____种痛苦。在总经____办公室，他惭愧

_____ 说，可能自己不____合这份工作。“安____工作吧，我会给____足够的时间，直____你成功为止。到____时，你再要走我____留你。”老总的宽____让年轻人很感____。他想，总该做出_____两件像样的事_____后再走。过了一_____，年轻人又走进_____老总的办公室。_____一次他是轻松_____，他已经连续 7_____月在公司销售_____行榜中高居榜____。原来，这份工作____那么适合他！
